# Lactate Production Precedes Inflammatory Cell Recruitment in Arthritic Ankles: an Imaging Study

**DOI:** 10.1007/s11307-020-01510-y

**Published:** 2020-06-08

**Authors:** Marie-Aline Neveu, Nicolas Beziere, Rolf Daniels, Caroline Bouzin, Arnaud Comment, Johannes Schwenck, Kerstin Fuchs, Manfred Kneilling, Bernd J. Pichler, Andreas M. Schmid

**Affiliations:** 1 Werner Siemens Imaging Center, Department of Preclinical Imaging and Radiopharmacy, Eberhard Karls University Tuebingen, Roentgenweg 13, 72076 Tuebingen, Germany; 2 Department of Pharmaceutical Technology, Eberhard Karls University Tuebingen, Tuebingen, Germany; 3 IREC Imaging Platform, Institute of Experimental and Clinical Research, Université catholique de Louvain, Brussels, Belgium; 4 General Electric Healthcare, Pollards Wood, Nightingales Lane, Chalfont St Giles, UK; 5 Department of Nuclear Medicine and Clinical Molecular Imaging, Eberhard Karls University Tuebingen, Tuebingen, Germany; 6 Cluster of Excellence iFIT (EXC 2180) “Image-Guided and Functionally Instructed Tumor Therapies”, University of Tuebingen, Tuebingen, Germany; 7 Department of Dermatology, Eberhard Karls University Tuebingen, Tuebingen, Germany

**Keywords:** Rheumatoid arthritis, Lactate, Hyperpolarized ^13^C-MRS, ^19^F-MRI, Immune imaging

## Abstract

**Purpose:**

Inflammation is involved in many disease processes. However, accurate imaging tools permitting diagnosis and characterization of inflammation are still missing. As inflamed tissues exhibit a high rate of glycolysis, pyruvate metabolism may offer a unique approach to follow the inflammatory response and disease progression. Therefore, the aim of the study was to follow metabolic changes and recruitment of inflammatory cells after onset of inflammation in arthritic ankles using hyperpolarized 1-^13^C-pyruvate magnetic resonance spectroscopy (MRS) and ^19^F magnetic resonance imaging (MRI), respectively.

**Procedure:**

Experimental rheumatoid arthritis (RA) was induced by intraperitoneal injection of glucose-6-phosphate-isomerase-specific antibodies (GPI) containing serum. To monitor pyruvate metabolism, the transformation of hyperpolarized 1-^13^C-pyruvate into hyperpolarized 1-^13^C-lactate was followed using MRS. To track phagocytic immune cell homing, we intravenously injected a perfluorocarbon emulsion 48 h before imaging. The animals were scanned at days 1, 3, or 6 after GPI-serum injection to examine the different stages of arthritic inflammation. Finally, to confirm the pyruvate metabolic activity and the link to inflammatory cell recruitment, we conducted hematoxylin-eosin histopathology and monocarboxylase transporter (MCT-1) immune histochemistry (IHC) of inflamed ankles.

**Results:**

Hyperpolarized 1-^13^C-pyruvate MRS revealed a high rate of lactate production immediately at day 1 after GPI-serum transfer, which remained elevated during the progression of the disease, while ^19^F-MRI exhibited a gradual recruitment of phagocytic immune cells in arthritic ankles, which correlated well with the course of ankle swelling. Histopathology and IHC revealed that MCT-1 was expressed in regions with inflammatory cell recruitment, confirming the metabolic shift identified in arthritic ankles.

**Conclusions:**

Our study demonstrated the presence of a very early metabolic shift in arthritic joints independent of phagocytic immune cell recruitment. Thus, hyperpolarized 1-^13^C-pyruvate represents a promising tracer to monitor acute arthritic joint inflammation, even with minor ankle swelling. Furthermore, translated to the clinics, these methods add a detailed characterization of disease status and could substantially support patient stratification and therapy monitoring.

**Electronic supplementary material:**

The online version of this article (10.1007/s11307-020-01510-y) contains supplementary material, which is available to authorized users.

## Introduction

Rheumatoid arthritis (RA) is an autoimmune disease affecting 1–2 % of the worldwide population and causes severe disabilities. The disease is characterized by a dense inflammatory cell infiltrate in the joints, proliferation of synovial cells, angiogenesis, and pannus formation. This chronic inflammation of the synovial tissues leads to cartilage and bone destruction, and ultimately to joint deformity and infirmity [1]. Thus, early diagnosis is essential to rapidly initiate anti-inflammatory treatment approaches with steroids, nonsteroidal anti-inflammatory drugs, or biologicals (anti-TNF mAbs, anti IL-17 mAbs) in order to limit tissue damage, especially in juvenile RA. The current diagnostic tools in patients rely on clinical examination, blood tests, radiography, power Doppler ultrasonography, computed tomography, or magnetic resonance imaging (MRI), but unfortunately lack specificity and are only capable of identifying rather advanced stages of arthritic joint diseases [2]. Consequently, there is a critical need for robust diagnostic methods that are able to identify early inflammatory processes within the inflamed joints, even before severe joint and cartilage destruction.

Metabolic reprogramming is a key feature of inflamed tissues, typically associated with reduced oxidation while favoring glycolysis and glutaminolysis [3], leading to high glucose demand, high lactate levels, and acidosis, directly contributing to cellular injury *via* proinflammatory signals [4, 5]. While early metabolic changes in RA and the effects of anti-inflammatory treatment approaches can be monitored by molecular imaging using the gold standard 2-deoxy-2-[^18^F]fluoro-D-glucose (^18^F-FDG) positron emission tomography (PET) [6–9], only two studies investigated lactate production in preclinical models of RA but exclusively examined the advanced severe stage using hyperpolarized 1-^13^C-pyruvate magnetic resonance spectroscopic imaging (MRSI) [10, 11]. In our studies, we examined the glucose-6-phosphate-isomerase (GPI)-serum-induced experimental RA to monitor the temporal dynamics of the effector phase of arthritic joint disease [12]. GPI-serum-induced experimental RA is independent of T and B cells, and is characterized by polymorphonuclear cell and macrophage recruitment specific to the sites of arthritic joints [13–15] and is best qualified to study the effector phase of acute arthritic joint disease, as it shares multiple similarities with the effector phase of RA in patients.

The aim of our study was to noninvasively monitor the transformation of hyperpolarized 1-^13^C-pyruvate into hyperpolarized 1-^13^C-lactate *in vivo* over time [16] and to determine its interplay with the temporal dynamics of phagocytic immune cell recruitment (phagocytes, such as macrophages) during the development of experimental RA with a perfluorocarbon (PFC) emulsion using ^19^F magnetic resonance imaging (^19^F-MRI) [17].

## Materials and Methods

### Animal Model of Arthritis

Acute GPI-serum-induced arthritis was induced as described previously [12, 18, 19]. Briefly, GPI-serum was obtained from generation 1 (K/BxN mice 1–3 months old) and diluted 1:1 (v/v) with saline before injection. Two hundred microliters of either diluted GPI-serum (*n* = 26) or saline (*n* = 11) was injected intraperitoneally into randomized groups of 8- to 12-week-old female BALB/c mice (Charles River GmbH, Erkrath, Germany). Control-serum was collected from healthy C57BL/6 mice. Caliper measurements of the ankle diameter (hind legs) were performed before serum transfer and at the indicated time points after serum transfer to follow ankle swelling.

Different cohorts of mice were examined with hyperpolarized ^13^C-MRS, ^19^F-MRI, or histology at days 1, 3, or 6 after GPI-serum injection to follow the early, moderate, and severe stages of inflammation in this model [18, 19]. Control animals were imaged on day 9 after saline injection.

MRS and MRI measurements were performed on a dedicated 7 T small animal MRI (BioSpec, Bruker BioSpin GmbH, Ettlingen, Germany) controlled by Paravision 6.0.1 (BrukerBioSpin GmbH). During experiments, the animals were anesthetized by the inhalation of isoflurane (Forene, Abbot, England) evaporated in 100 % O_2_ and warmed using a circulating water system. All applicable institutional and/or national guidelines for the care and use of animals were followed.

### Hyperpolarized ^13^C-MRS

For hyperpolarized ^13^C-MRS measurements, the animals were fasted overnight. 1-^13^C-pyruvate solutions were hyperpolarized using a DNP polarizer (SpinLab, General Electric, Waukesha, USA). C1-labeled pyruvic acid (Cortecnet, Voisins-le-Bretonneux, France) was polarized according to the manufacturer’s instructions. After 60–90 min, the hyperpolarized sample was rapidly dissolved and neutralized in an aqueous solution containing 40 mMTris, 60 mMNaOH, and 0.1 g/L Na_2_EDTA. The hyperpolarized 1-^13^C-pyruvate solution (80 mM) was intravenously injected through a catheter placed in the tail vein of the animal.

Both hind legs of the animal were positioned on a ^1^H/^13^C surface coil (2 cm in diameter, Bruker BioSpin GmbH). Started directly before the injection of the hyperpolarized 1-^13^C-pyruvate solution, coil-localized ^13^C spectra of the ankles were acquired every second for 240 s using a single pulse sequence (Bandwidth: 3019.32 Hz; α: 5°; 1024 points).

Using jMRUI v5, a zero-order phase correction was manually applied, and a 50 Hz apodization filter was executed on the time series. The data were then analyzed based on a model free-approach using the lactate-to-pyruvate ratio (Lac/Pyr) [20] calculated from the total areas under the dynamic curves of the 1-^13^C-lactate and 1-^13^C-pyruvate phased signals.

Apparent decay constants were fit to the decreasing part of the normalized peak using a mono-exponential function (Prism 7, GraphPad Software, San Diego, CA, USA). Values where a 95 % confidence interval could not be provided were excluded from the analysis (*n* = 3).

### ^19^F-MRI

For generation of the ^19^F-PFC emulsion, 10 % wt/wt perfluoro-15-crown-5 ether (Fluorochem, Derbyshire, UK) and 4 % wt/wt purified egg lecithin E80S (Lipoid, Ludwigshafen, Germany) were mixed in isotonic buffer (10 mM HEPES, 2.5 % glycerol, pH 7.4) to yield a coarse raw emulsion [21]. This raw emulsion was homogenized with a high-pressure (75 MPa, 10 cycles) Emulsiflex C5 homogenizer (Avestin, Mannheim, Germany). A final particle size of 110 ± 5 nm was achieved, as determined by dynamic light scattering using a Zetasizer Nano-ZS (Malvern Instruments Ltd., Malvern, UK).

The ^19^F-PFC emulsion was administered intravenously to the animals at 48 h before ^19^F-MRI detection using an infusion pump (100 μl/min for 5 min) to track the accumulation of phagocytic cells at the site of inflammation.

Animals were placed in a ^1^H-^19^F transmit-receive volume coil (inner diameter of 40 mm, Bruker BioSpin GmbH).^19^F axial images of the hind legs were obtained using a rapid acquisition with relaxation enhancement (RARE) sequence (repetition time (TR), 3000 ms; echo time (TE), 7.65 ms; RARE factor, 17; field of view (FOV), 40 × 40 mm^2^; slice thickness, 2 mm; number of averages (NA), 180; acquisition time, 9 min). ^1^H reference images were obtained using a T2-weighted 3-dimensional RARE sequence (TE/TR, 30.82/800 ms; RARE factor, 16; FOV, 60 × 32 × 23 mm^3^). The ^1^H/^19^F MRI images were fused and analyzed using PMOD 3.2 software (PMOD Technologies Ltd., Zurich, Switzerland). The ^19^F signal was determined similarly to the method presented by Temme *et al* [21]. The 2D regions of interest (ROIs) were drawn on consecutive axial slices covering the legs from palms to knees. The integrated intensity within the ROIs was then calculated for each leg of the animals. Overlays of the ^1^H/^19^F images were generated using Inveon Research Workplace software (Siemens Healthineers, Erlangen, Germany).

### Histology and Immunohistochemistry

After sacrifice, both hind limbs were dissected and fixed immediately for 24 h in 4 % paraformaldehyde. Tissue samples were then decalcified in 10 % EDTA for 10 days, followed by 3 days in a decalcifying solution (OsteoRAL R fast decalcifier, VWR International Ltd., Leicestershire, UK) at room temperature with continuous agitation before paraffin embedding. The tissues were cut into 5 μm sections before staining. Hematoxylin and eosin (H&E) staining was performed following standard procedures. Tissue sections were also stained for monocarboxylate transporter 1 (MCT-1) (rabbit anti-MCT1, Sigma-Aldrich, St. Louis, USA). The DakoEnVision+ anti-rabbit kit (Agilent Technologies, Santa Clara, USA) was used to detect primary antibodies. Stained slides were digitalized using a SCN400 slide scanner (Leica Biosystems, Wetzlar, Germany).

### Statistical Analysis

Analysis was performed using GraphPad Prism 7 software. The results are expressed as the mean value ± SEM. One-way ANOVA with Dunnett’s test *post hoc* was performed to investigate any mean difference over time of thickness in arthritic or control ankles. An unpaired t-test was used to compare the mean signal changes in the arthritic and control ankles. All statistical tests were two-sided, with *p* < 0.05 (*), *p* < 0.01 (**), or *p* < 0.001 (***) considered significant.

## Results

### Lactate Production Peaks before Maximal Joint Swelling

**Fig. 1:**
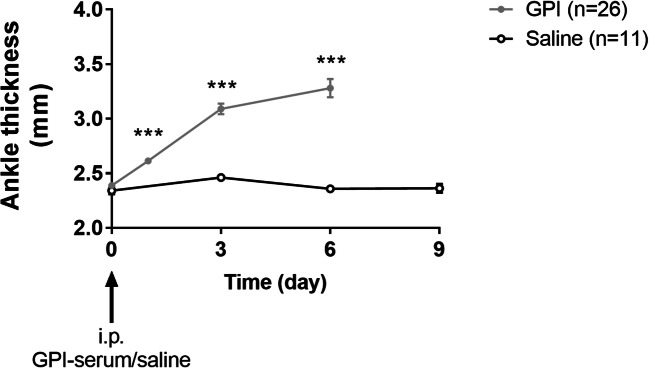
Course of ankle swelling. A significant increase in ankle thickness was observed in GPI-serum injected mice (*n* = 26), while ankles of saline injected animals (*n* = 11) remained unaffected (*p* < 0.001). Hyperpolarized ^13^C-MRS and ^19^F-MRI were performed on different cohorts of animals at day 1, day 3, or day 6 after GPI-serum injection (indicated by gray arrows) to follow the early and severe stages of inflammation in this model. Control animals were scanned at day 9 after saline injection (indicated by a black arrow). Data are expressed as the mean ± SEM. One-way ANOVA was used to follow the course ankle swelling in each group (each time point compared with day 0).

Hyperpolarized ^13^C-MRS was performed on different cohorts of animals on days 1, 3, or 6 after GPI-serum injection to examine lactate production during the early and severe stages of inflammation in this acute model of arthritis (Fig. 1). A significant increase in ankle thickness was already observed after day 1 in GPI-serum-injected mice compared with baseline. This effect was followed by the expansion of the swelling until day 6 (*n* = 26; *p* < 0.0001). The ankles of saline-injected animals remained unaffected (*n* = 11; *p* = 0.1378). All animal cohorts used for this study demonstrated similar ankle swellings as previously reported [18, 19].

**Fig. 2:**
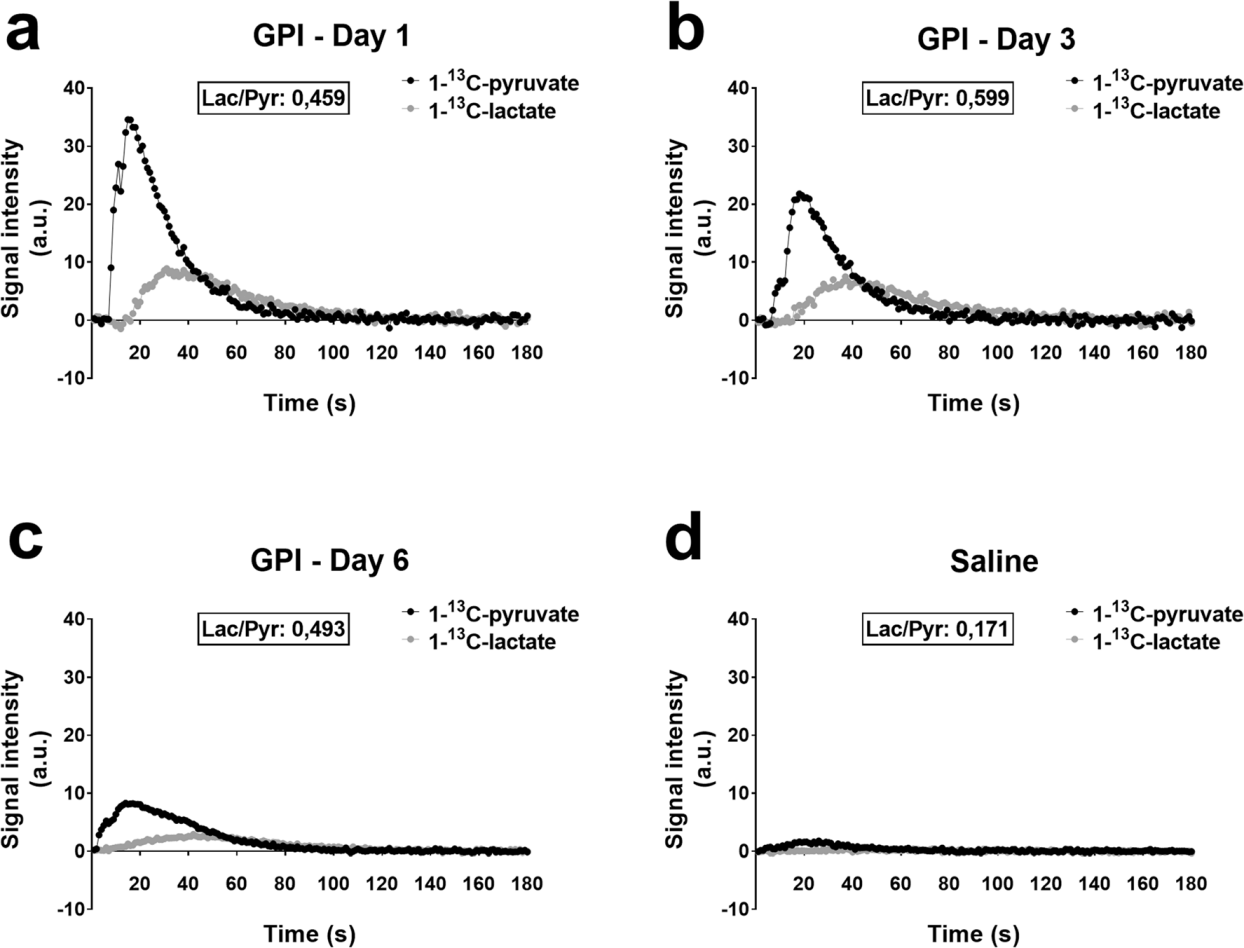
Hyperpolarized 1-^13^C-pyruvate-MRS reveals lactate formation in arthritic ankles. Representative pyruvate and lactate time intensity curves after i.v. injection of hyperpolarized 1-^13^C pyruvate from representative arthritic (a–c) and control (d) ankles. The lactate-to-pyruvate (lac/Pyr) ratio, representing lactate production, was quantified based on the areas under the curves of the 1-^13^C-lactate and 1-^13^C-pyruvate signal intensities. A.u., Arbitrary units.

**Fig. 3:**
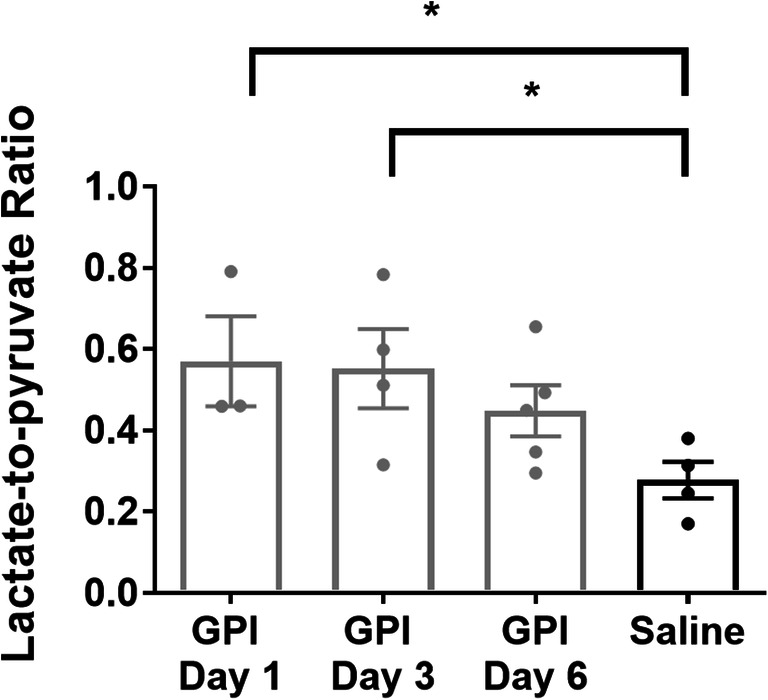
Quantification of 1-^13^C-pyruvate transformation into 1-^13^C-lactate reveals an early metabolic shift occurring in arthritic ankles. Lactate production, measured by the lactate-to-pyruvate ratio, is at the highest level early in the disease (GPI day 1) and decreases with later stages. Individual values per animal are displayed as dots. Data are expressed as the mean ± SEM. Unpaired t-tests were two-sided. *n* = 3–5 mice/group. A.u., Arbitrary units.

Evolution of lactate signal in the ankles typically differentiates arthritic (Fig. 2a–c) and control (Fig. 2d, Supplemental Fig. S1) mice during hyperpolarized 1-^13^C-pyruvate MRS experiments. In GPI-serum-injected animals, the lactate signal was visibly increased at all time points and correlated with a higher pyruvate signal, while both signals remained at baseline in saline-injected animals. After GPI-serum transfer, an elevated Lac/Pyr ratio, reflecting lactate production, was immediately identified at day 1 (Fig. 3). The mean Lac/Pyr ratio shifted from 0.57 ± 0.11 at day 1 (*n* = 3; *p* = 0.0407) to 0.55 ± 0.10 at day 3 (*n* = 4; *p* = 0.0429) and to 0.45 ± 0.06 at day 6 (*n* = 5; *p* = 0.0743) when compared with control ankles, exhibiting a ratio of 0.23 ± 0.04 (*n* = 4). Interestingly, lactate production peaked as early as 1 day after the GPI-serum transfer when hardly any ankle swelling was detectable. Notably, the time-to-peak for lactate production was fastest at day 1 and increased with disease progression (Fig. 2, Supplemental Fig. S2); however, this effect was not significant compared with the control group. The apparent decays from the average of all RA (control) spectra were 24.0 ± 1.9 s (18.5 ± 1.2 s) and 46.6 ± 2.1 s (26.4 ± 6.6 s) for hyperpolarized 1-^13^C-Pyruvate and hyperpolarized 1-^13^C-Lactate, respectively.

### Immune Cell Recruitment Follows the Course of Ankle Swelling

**Fig. 4:**
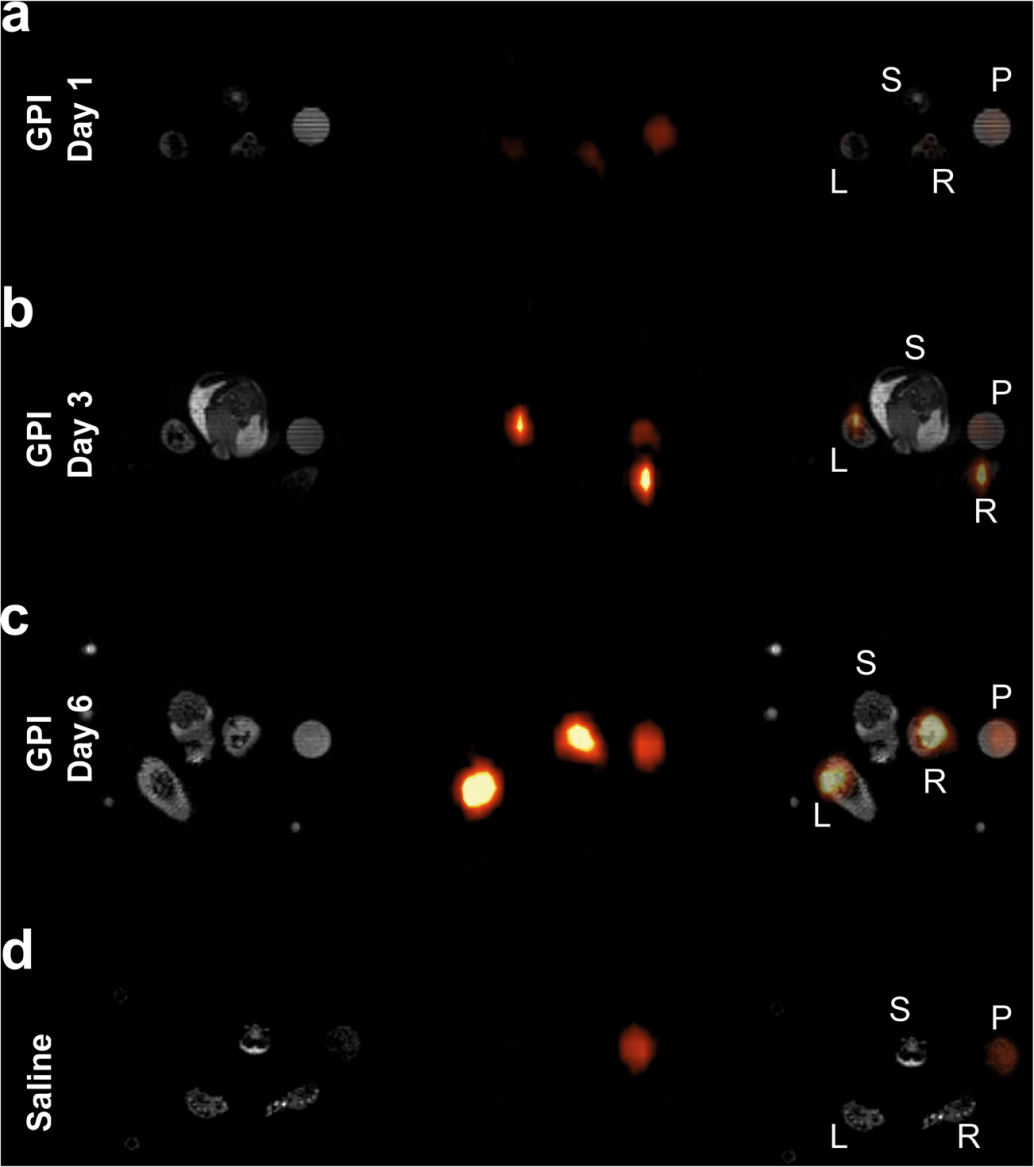
^19^F-MRI exhibits PFC accumulation in arthritic ankles of GPI-serum injected mice. Representative anatomical ^1^H images (grayscale), ^19^F images (pseudocolor) and merged ^1^H/^19^F images for visualization PFC accumulation into arthritic (a–c) and control (d) ankles. The PFC emulsion was injected 2 days prior to imaging. ^19^F axial slices are identically scaled. Labels are S = spine, P = reference phantom, L = left leg, R = right leg.

**Fig. 5:**
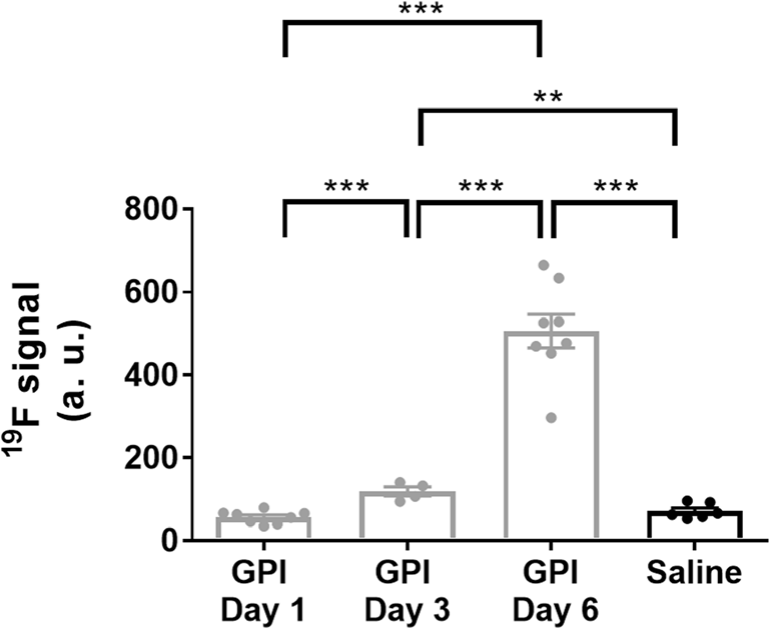
Quantification of the ^19^F signal highlights progressive immune cell recruitment in arthritic ankles. Measurements of individual legs are presented. While at day 1 macrophage levels in arthritic ankles are comparable to the control ankles, a continuous increase in ^19^F-PFC signal is evident following ankle swelling. Data are expressed as the mean ± SEM. Unpaired t-tests were two-sided. *n* = 2–4 mice/group. A.u., Arbitrary units.

To identify a link between the dynamics of lactate production and immune cell recruitment in our model, ^19^F-MRI measurements were performed after the injection of a PFC emulsion on a different cohort of animals. Subsequently, ^19^F images were fused with corresponding ^1^H anatomical images of arthritic (Fig. 4a–c) and unaffected naïve (Fig. 4d) ankles of GPI-serum-injected or healthy experimental mice. Merged images revealed a clear PFC accumulation in both paws of arthritic animals at day 3 (Fig. 4b) and day 6 (Fig. 4c). Twenty-four hours after the onset of GPI-serum-induced arthritis, we observed no enhancement of the ^19^F signal (56.7 ± 5.3; *n* = 4; *p* = 0.1254), whereas on day 3, a significant increase was observed in the arthritic ankles (118.6 ± 10.8; *n* = 2; *p* = 0.0056) compared with the healthy control ankles (71.3 ± 7.4; *n* = 3) (Fig. 5). On day 6, a 7.1-fold enhancement of the ^19^F signal was observed in the arthritic ankles (506.3 ± 40.3; *n* = 4; *p* < 0.0001) when compared with the ankles of healthy mice. In arthritic ankles, a gradual increase of the ^19^F signal was observed between day 1, day 3 (2.1-fold increase between day 1 and day 3; *p* = 0.0002), and day 6 (8.9-fold increase between day 1 and day 6; 4.3-fold increase between day 3 and day 6; *p* < 0.0001 both). The quantification of the ^19^F signal in the ankles reveals a progressive immune cell recruitment in arthritic animals that correlates with the course of ankle swelling.

### MCT-1 Staining Supports an Early Metabolic Shift Identified in Arthritic Ankles

**Fig. 6:**
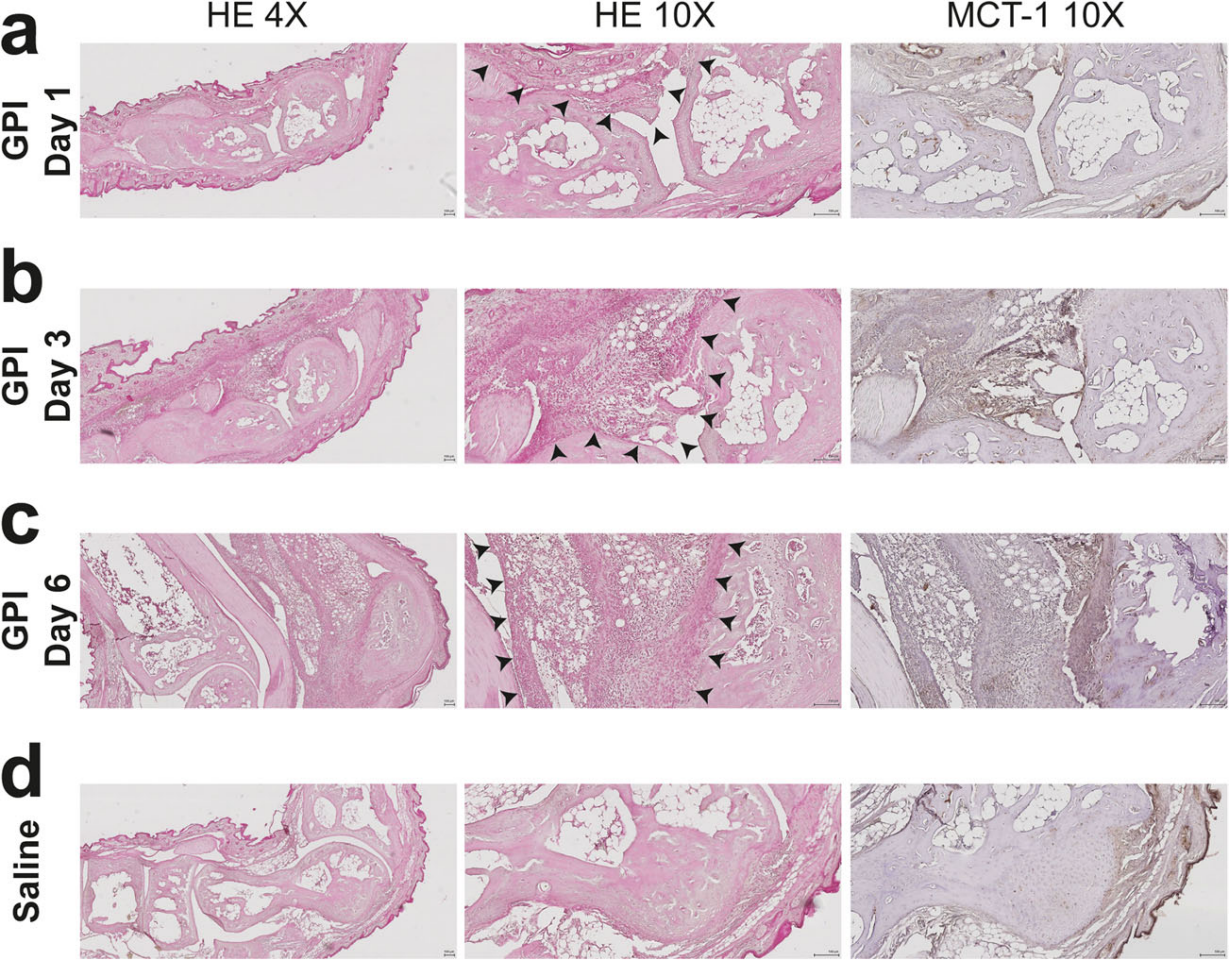
Histological analysis confirms leucocyte accumulation and enhanced metabolic activity in arthritic ankles. Representative H&E and MCT-1 stained slices of arthritic (a-c) and control (d) ankles. While no signs of inflammation were identified in control ankles, arthritic ankles revealed increasing inflamed regions (indicated by arrowheads) on H&E stained slices (left and center panels). MCT-1 staining (right panels) demonstrated increased enzyme expression in the inflamed regions of arthritic ankles.

To confirm the inflammatory response and the link with pyruvate metabolic activity in synovial membrane tissues of arthritic ankles, the expression of the MCTs was evaluated in parallel with H&E staining (Fig. 6). The initiation of histological changes, including immune cell infiltration and hyperplasia of the synovial membrane, was identified at day 1 after GPI-serum administration (Fig. 6a, left-hand side and center), with a progression of pannus formation until day 6. At this stage of the disease, a dense immune infiltration was observed in the synovium and the subsynovial pericapsular tissue (synovitis) (Fig. 6c, left-hand side and center). Strong periarthritis was also observed in the subcutaneous tissue adjacent to the joint. These observations were consistent with our previous studies on this model [18] and correlated with the *in vivo*^19^F-MRI results. Immunohistochemical staining showed limited MCT-1 expression in the control ankles with staining patterns in the skin and the subcutaneous tissue adjacent to the joint (Fig. 6d, right-hand side). In the arthritic ankles (Fig. 6a–c, right-hand side), the regions with inflammatory cell recruitment, such as the synovitis and the periarthritic regions, expressed MCT-1 and correlated with the severity of the disease supporting the presence of a high metabolic activity in the inflamed regions. As MCT-1 is responsible for the transport of hyperpolarized 1-^13^C-pyruvate and has been characterized as one of the limiting factors for its kinetic conversion into lactate [22, 23], the limited expression of MCT-1 in control ankles reflects the restricted pyruvate signal and lactate conversion observed by ^13^C-MRS (Fig. 2d, Fig. 3) and confirms the metabolic shift identified in arthritic ankles (Fig. 2a–c, Fig. 3).

## Discussion

By following the course of lactate production during the progression of arthritis, we identified an early peak of lactate that was associated with limited immune cell infiltration in arthritic ankles. Our previous imaging studies on GPI-serum-induced arthritis revealed that the course of ankle swelling was associated with increased cellular proliferation, followed by 3′-deoxy-3′-[^18^F]-fluorothymidine (^18^F-FLT) PET [18]. Additionally, [^18^F]-fluoromisonidazole (^18^F-FMISO) and [^18^F]-fluoroazomycinarabinoside (^18^F-FAZA) PET probes revealed an early onset of hypoxia at day 3 post-GPI-serum transfer [19] that was associated with enhanced α_v_β_3_ integrin activity, as determined by [^18^F]-galacto-RGD PET [12], revealing angiogenesis in the diseased ankles. Finally, reactive oxygen species (ROS) stress was identified as maximal during the severe phase of the disease at day 6 [19] by using a ROS-sensitive chemiluminescence optical imaging probe L-012. As ROS are mainly generated by neutrophils and macrophages during inflammation, both cell types are critically involved in experimental GPI-serum-induced arthritis [14, 15, 24], we hypothesized that the progression of the disease could be driven by the progressive recruitment of active inflammatory cells into the joints, leading first to hypoxia and finally to ROS production. We wanted to correlate immune cell recruitment with the presence of a glycolytic phenotype in the joints of this experimental RA model. Interestingly, our studies revealed that an early metabolic shift uncovered by the determination of increased lactate production in inflamed ankles precedes pronounced phagocytic immune cell recruitment.

The metabolic shift occurring during joint inflammation has been previously revealed using molecular imaging such as ^18^F-FDG PET and hyperpolarized 1-^13^C-pyruvate MRSI [10, 11]. In a nonsystemic antigen-independent model of local severe experimental RA induced by a single subcutaneous injection of complete Freund’s adjuvant (CFA) into the hind paws, MacKenzie and colleagues assessed alterations in the conversion of hyperpolarized 1-^13^C-pyruvate into 1-^13^C-lactate, suggesting that this new tool may be used to indicate the presence of inflammatory arthritis [10]. Inflamed rat paws exhibited a Lac/Pyr ratio of 0.52 ± 0.16 (mean ± SD) compared with control paws presenting a Lac/Pyr ratio of 0.32 ± 0.11. More recently, Wright and coworkers confirmed these results in CFA-induced joint inflammation in mice [11]. A higher Lac/Pyr ratio of 0.74 ± 0.32 (mean ± SD) was found in inflamed joints when measuring the entire signal coming from the inflamed feet of a small cohort of animals. While the results of both studies correlated with previous reports of increased lactate levels in the synovial fluid of RA patients [25, 26], only the severe phase of the arthritic joint disease was investigated when the joint swelling was maximal. Our hyperpolarized ^13^C MRS data were consistent with the values reported by MacKenzie *et al* and Wright *et al* [10, 11]. We identified a Lac/Pyr ratio of 0.45 ± 0.06 (mean ± SEM) in arthritic ankles during the severe phase at day 6 (Fig. 3). This effect was associated with a high ^19^F-signal in the ankles (Fig. 5), showing a massive immune cell infiltrate that correlates with the swelling and the severity of the inflammation. However, when focusing on other time points, such as the early and moderate phase of the disease, we identified that lactate production in inflamed joints was higher than that during the severe phase, with an increased Lac/Pyr ratio at day 1 (0.57 ± 0.11) (Fig. 3). Surprisingly, the enhanced lactate production in inflamed ankles did not correlate with a pronounced phagocytic immune cell infiltrate, as the ^19^F-signal increased delayed at day 3 after the onset of experimental RA (Fig. 5).

While the ^19^F-signal followed the course of ankle swelling and reflected our previous data where we identified a gradual increase of proliferation in the arthritic ankles using ^18^F-FLT [18], highlighting the progressive immune cell infiltration during the progression of inflammation, lactate production was highest during the early stage of the disease, which was associated with limited immune cell recruitment. While synovial lactate levels are increased in patients with established RA [25, 26], our study noninvasively identified that lactate production is already increased during the onset of the disease. Thus, noninvasive determination of lactate production might represent a novel diagnostic tool opening a window of opportunity for immediate anti-inflammatory treatment at very early stages of RA, which is of utmost importance for long-term remission of RA [27]. Furthermore, our results showed that the glycolytic phenotype observed in the joints does not originate only from macrophages and neutrophils only and therefore also other cell types may undergo metabolic reprogramming during the disease. Among these, fibroblast-like synoviocytes (FLSs), normally resident into the joints, have recently been demonstrated to play a key role in both the initiation and perpetuation of the disease after activation [28]. Activated FLSs acquire a tumor-like and aggressive phenotype associated with metabolic reprogramming, as well as new functions, such as chemokine and cytokine production [28, 29], thus playing an active role in immune cell recruitment and bone distortion [30]. While further investigations of the role and behavior of FLSs during the early phase of the disease are necessary, our results strongly indicate the activity of resident cells such as FLS. Recently, SPECT and PET probes were described to study the impact of fibroblasts in RA by targeting the fibroblast activation protein (FAP) and displayed uptake into slightly inflamed joints, while ^18^F-FDG uptake was only able to reveal severely inflamed joints in mice. Used in combination with technologies targeting immune cell dynamics, the role of all cell actors involved in the pathogenesis of RA could be revealed and linked to variations of metabolic activities occurring during disease evolution.

Given the growing use of hyperpolarized 1-^13^C-pyruvate MRSI in patients [31], this technology could represent a valuable tool to noninvasively identify and monitor joint inflammation compared with invasive synovial fluid samplings or nonspecific blood tests, especially for juvenile patients or patients only partially fulfilling the classification criteria of RA.

## Conclusion

By combining for the first time hyperpolarized 1-^13^C-pyruvate MRS for metabolic profiling and ^19^F-MRI for phagocytic immune cell tracking, we were able to noninvasively demonstrate the presence of an early metabolic shift in arthritic ankles independently of phagocytic immune cell recruitment. In addition to the early diagnosis of RA, the combination of these novel technologies, potentially including [^18^F]FLT-PET [19], could represent a unique platform to characterize disease stages by revealing metabolic perturbations before joint damage. Furthermore, this improves the monitoring of anti-inflammatory treatment approaches and could thereby limit the number of patients disabled by joint destruction.

## Electronic supplementary material

ESM 1 (DOCX 80 kb)
