# Supplementary material for: Lactate Production Precedes Inflammatory Cell Recruitment in Arthritic Ankles: an Imaging Study
Source: Mol Imaging Biol. 2020 Jun 8;22(5):1324–32. doi: 10.1007/s11307-020-01510-y (PMC7497460; doi:10.1007/s11307-020-01510-y)
Supplement: Supplementary file 1 — (DOCX 80 kb) [file 11307_2020_1510_MOESM1_ESM.docx]

Supplemental Information to

**Lactate production precedes inflammatory cell recruitment in arthritic ankles: an imaging study**

Marie-Aline Neveu^1^, Nicolas Beziere^1^, Rolf Daniels^2^, Caroline Bouzin^3^, Arnaud Comment^4^, Johannes Schwenck^1,5,7^, Kerstin Fuchs^1^, Manfred Kneilling^1,6,7^, Bernd J. Pichler^1,7^ and Andreas M. Schmid^1^

Figure S1:


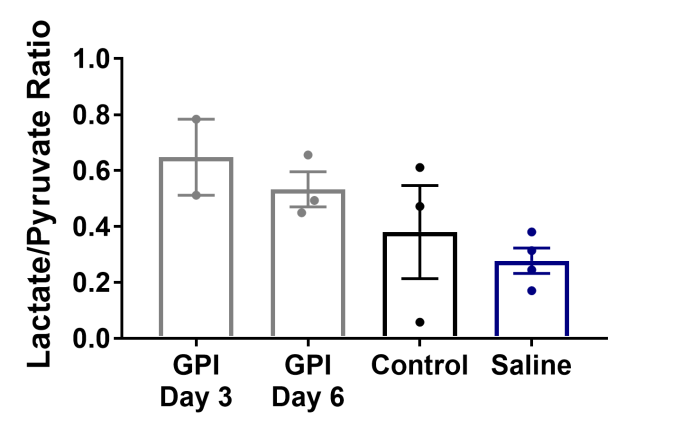


Figure S1: In a pilot study, GPI-serum, control-serum from healthy C57BL/6 mice or saline was injected into BALB/c mice. GPI-serum injected mice showed increased Lactate/Pyruvate Ratios in the ankles at days 3 and 6 post serum injection using hyperpolarized ^13^C-MRS, while control-serum and saline-injected animals showed comparable Lactate/Pyruvate Ratios in the ankles 9 days post injection. As the saline data showed reduced standard deviations and no significant difference to the control serum, we used saline as reference values.

Figure S2:


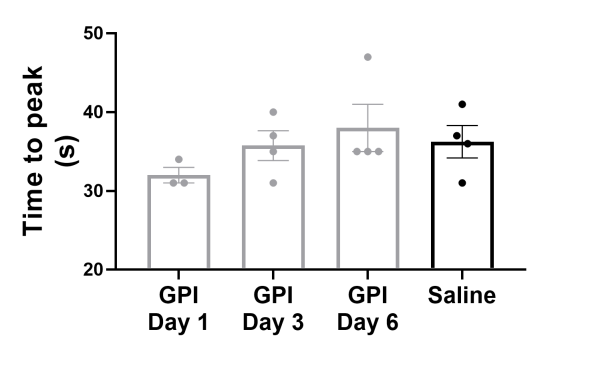


Figure S2: Kinetic Analysis: Time-to-Peak of the lactate production over the course of disease progression. Lactate peaked earliest on day1 after GPI serum transfer and slowed down to control level over the time course of the experiments.
